# Epigenetic regulation in tomato fruit ripening

**DOI:** 10.3389/fpls.2023.1269090

**Published:** 2023-09-14

**Authors:** Yucheng Ming, Libo Jiang, Dongchao Ji

**Affiliations:** School of Life Sciences and Medicine, Shandong University of Technology, Zibo, China

**Keywords:** tomato, fruit ripening, epigenetic regulation, molecular mechanisms, regulatory network

## Abstract

Fruit ripening is a crucial stage in quality development, influenced by a diverse array of internal and external factors. Among these factors, epigenetic regulation holds significant importance and has garnered substantial research attention in recent years. Here, this review aims to discuss the breakthrough in epigenetic regulation of tomato (*Solanum lycopersicum*) fruit ripening, including DNA methylation, N^6^-Methyladenosine mRNA modification, histone demethylation/deacetylation, and non-coding RNA. Through this brief review, we seek to enhance our understanding of the regulatory mechanisms governing tomato fruit ripening, while providing fresh insights for the precise modulation of these mechanisms.

## 1 Introduction

As a unique organ of angiosperms, fruit provides rich dietary fiber, vitamins and other nutrients for human beings, and is an important component of healthy dietary structure (Giovannoni, 2001; Chen et al., 2020). Fruit ripening is a key period for the formation of fruit edible quality, which is a complex developmental process involves changes in fruit texture, pigment accumulation, formation of aroma and flavor substances, reduction of resistance and other traits, and regulated by many internal and external factors (Giovannoni, 2004; Ji and Wang, 2023). The internal factors mainly include transcription factors and hormones, while the external factors mainly include various biological factors and abiotic factors. According to the different respiratory patterns, fruits can be divided into two types: climacteric and non-climacteric (Mcmurchie et al., 1972). During the ripening process, the respiratory intensity and ethylene release of climacteric fruits appeared the accompanied burst, such as tomato, apple and banana. However, there was no significant change in respiratory intensity and ethylene release in non-climacteric fruits, such as strawberry, grape, citrus (Shinozaki et al., 2018). Two systems of ethylene biosynthesis (System I and System II) play vital roles in the development and ripening of climacteric fruits. Immature fruits and other organs of the plant continuously produce low concentrations of ethylene, that is, the background concentration of ethylene. System I ethylene regulates ethylene synthesis of background concentration in a negative feedback way and participates in fruit development, while system II ethylene is produced in an autocatalytic manner during fruit ripening (Liu M. et al., 2015). The consensus view asserts that ethylene usually collaborates with transcription factors to collectively orchestrate the regulation of fruit ripening (Li et al., 2022). In tomato, MADS-box transcription factors are recognized as key transcriptional regulators that govern fruit ripening (Smaczniak et al., 2012; Gapper et al., 2013). Among these, the MADS-box transcription factor RIPENING-INHIBITOR (RIN) plays a crucial role in the regulation of tomato fruit ripening and is considered a master regulator (Vrebalov et al., 2002; Li et al., 2019). Researchers have employed Chromatin Immunoprecipitation (ChIP) techniques to progressively identify a series of target genes regulated by RIN. These target genes are involved in various aspects of fruit ripening, including ethylene biosynthesis and signaling (Ito et al., 2008; Li et al., 2011), fruit softening (Fujisawa et al., 2011), pigment synthesis (Martel et al., 2011), aroma compound synthesis (Qin et al., 2012), protein ubiquitination (Wang et al., 2014), and sucrose metabolism (Qin et al., 2016). TOMATO AGAMOUS-LIKE1 (TAGL1) is another member of the MADS box family. In tomato, silencing of *TAGL1* inhibits the fruit ripening process, leading to thinner fruit skin, reduced ethylene release, and an incomplete transition to red coloration (Vrebalov et al., 2009). It was reported that TAGL1 can regulate ethylene biosynthesis by activating the expression of the *ACS2* gene (Itkin et al., 2009). FRUITFULL1 (FUL1) and FUL2 are also members of the MADS box family of transcription factors, and both proteins can interact with RIN (Bemer et al., 2012). Simultaneous silencing of both genes led to a reduction in tomato lycopene content (Bemer et al., 2012). FUL1/FUL2 target over 800 genes, with some overlapping with RIN target genes. *In vitro* experiments have indicated that FUL1, FUL2, RIN, and TAGL1 may form a quaternary complex to collectively regulate fruit ripening (Bemer et al., 2012; Fujisawa et al., 2014). In the context of tomato fruit, RIN and TAGL1 form a complex that triggers the activation of ethylene biosynthesis genes, thereby establishing a positive feedback loop that generates autocatalytic ethylene (Lü et al., 2018; Ji et al., 2020). This MADS-type circuit subsequently stimulates downstream ripening-associated genes.

Tomato (*Solanum lycopersicum*), an important economic crop, is cherished for its delicious and nutritious fruit. With a relatively small genome size (~900 Mb), well-defined genetic background, abundant mutant resources, and ease of genetic transformation, the tomato has emerged as a model plant for studying the biology of climacteric fruit development (Giovannoni et al., 2017; Liu et al., 2022). Additionally, the distinct stages of fruit ripening, coupled with its evident attributes, make it an ideal candidate for investigating the biology of climacteric fresh fruit ripening. A large number of studies have focused on transcription and ethylene regulation of tomato fruit ripening. Due to the rapid development of new sequencing technologies, the study of epigenetics has greatly promoted the molecular mechanism of tomato fruit ripening (Giovannoni et al., 2017). An increasing body of evidence suggests that epigenetic mechanisms play a role in modulating the process of tomato fruit ripening, and the regulatory mechanisms of some important proteins and genes have been analyzed (Zhong et al., 2013; Giovannoni et al., 2017; Liu et al., 2022). Epigenetics refers to heritable changes in gene expression without altering the original gene sequence (Eichten et al., 2014; Ji and Wang, 2023). Epigenetic regulation encompasses diverse mechanisms, including methylation modification of DNA or RNA, histone modification, non-coding RNA. Here, this review provides an overview of the breakthrough and future prospects for studies of epigenetic regulation in tomato fruit ripening.

## 2 Epigenetic regulation of tomato fruit ripening

### 2.1 DNA/RNA methylation

DNA methylation referred a conserved process involving the transfer of a −CH3 group to the fifth carbon of cytosine residues, resulting in the formation of 5-methylcytosine (5mC), exerting a significant impact on chromatin function, usually down-regulating gene expression (Zhang et al., 2018; He et al., 2022). A classic example of methylation-mediated regulation in plant fruit ripening arises from investigations of the tomato *cnr* (*Colorless non-ripening*) mutants. *CNR* was located on tomato chromosome 2, belonged to the SBP (squamosa promoter binding protein) family of transcription factors. Compared with the wild type, ethylene synthesis, fruit softening and carotenoid synthesis of *cnr* mutant fruits were inhibited (Eriksson et al., 2004). At the same time, the fruit texture of the mutant was loose, the intercellular adhesion was decreased, and the fruit was easy to crack. It was found that *CNR* DNA sequence was not changed in *cnr* mutants, while the promoter region showed hypermethylation, and CNR transcription was inhibited (Manning et al., 2006). Nonetheless, recent studies have indicated that following the application of CRISPR/Cas9 gene editing technology to knock out *CNR*, there was no substantial alteration observed in the fruit ripening phenotype in comparison to the wild type. Furthermore, the fruit phenotype associated with the *cnr* mutant could not be replicated (Gao et al., 2019). Therefore, the molecular regulation mechanism of this transcription factor still needs further study.

**Figure 1:**
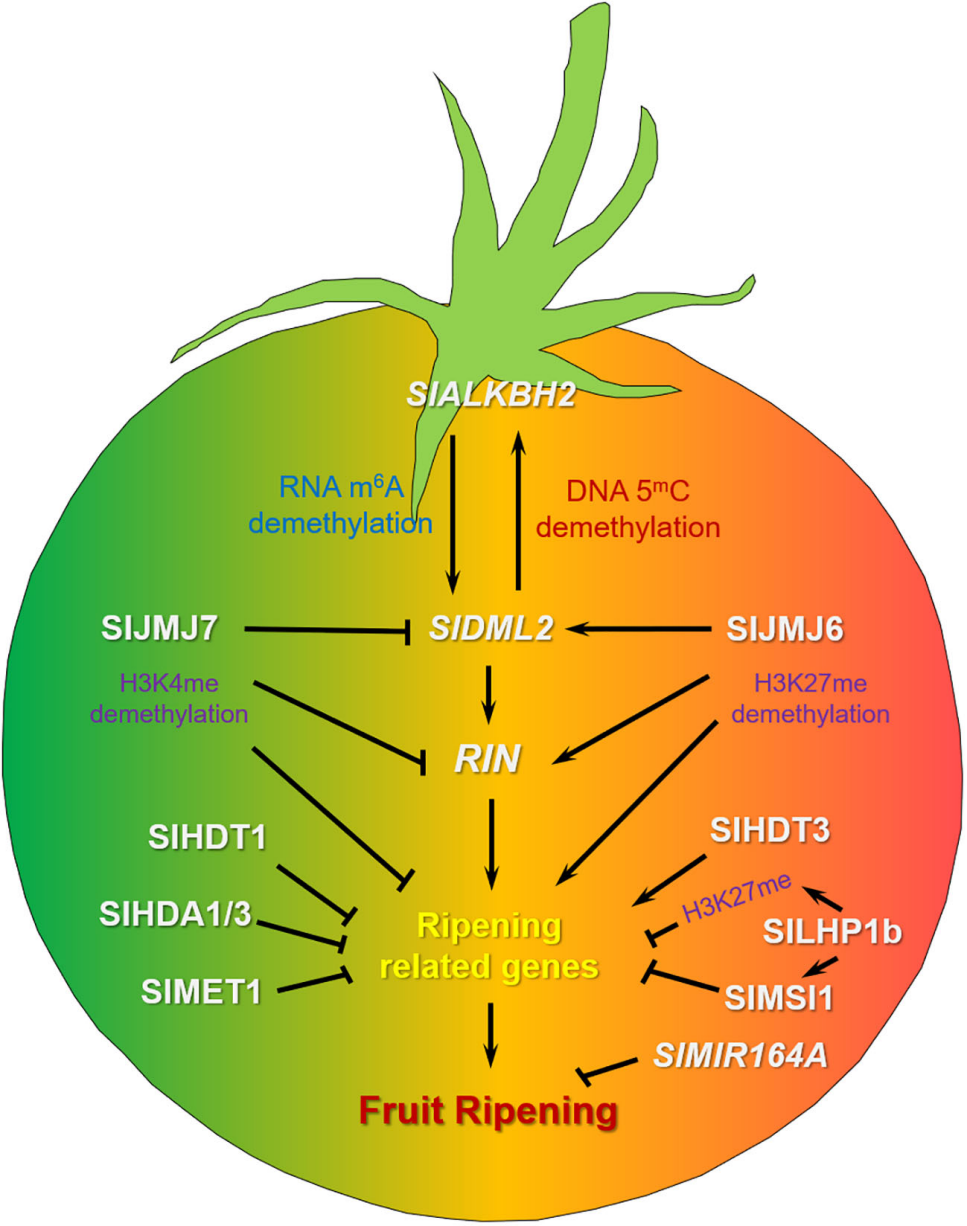
The regulation network of DNA methylation, RNA methylation, histone modification and non-coding RNA in tomato fruit ripening. *SlALKBH2* regulates the stability of *SlDML2* RNA by RNA m^6^A demethylation, enhancing its stability. Conversely, *SlDML2* promotes the expression of *SlALKBH2* through DNA demethylation, ultimately affecting fruit ripening. The histone demethylase SlJMJ6 eliminates H3K27me marks from *SlDML2*, *RIN* and ripening-associated genes, consequently promoting fruit ripening. On the other hand, histone demethylase SlJMJ7 erases H3K4me modifications from these genes, thus contributing to the inhibition of fruit ripening. Tomato methyltransferase SlMET1, Histone deacetylases SlHDA1, SlHDA3 and SlHDT1 may negatively regulate ripening-associated genes to control fruit ripening, while SlHDT3 exhibits opposing regulatory effects. PRC1 protein SlLHP1b could bind H3K27me mark in regions of ripening-associated chromatin, targeting ripening-related genes, repressing fruit ripening. Meanwhile, SlLHP1b interacted with PRC2 protein SlMSI1, negatively regulating tomato fruit ripening. The microRNA gene *SlMIR164A* was involved in negative regulation of tomato fruit ripening.

To determine the intrinsic link between DNA methylation and fruit ripening, researchers treated tomato fruits with methyltransferase inhibitor 5-azacytidine and found that the ripening process was accelerated, while hypermethylation was observed in the *cnr* and *rin* (*ripening inhibitor*) mutants fruits (Zhong et al., 2013). Further genome-wide methylation sequencing found that DNA methylation levels decreased continuously as the fruit matured (Zhong et al., 2013). The results indicated that DNA methylation was a dynamic process during different fruit ripening stages. Functional analysis of four DNA DEMETER-like DNA demethylases (DMLs) in tomato showed that *SlDML2* was highly expressed in fruit and showed ripening related expression patterns (Liu R. et al., 2015). After this gene was silenced or knocked out, tomato fruit ripening process was delayed, indicating that DNA demethylase played an important role in the regulation of fruit ripening (Liu R. et al., 2015; Lang et al., 2017). A recent study found that *SlDML2* not only regulated *RIN* expression, but also regulated RIN binding in tomato fruit ripening process (Niu et al., 2022). In addition, studies have shown that RNA demethylase *Solanum lycopersicum* AlkB homolog 2 (SlALKBH2) can bind the mRNA of *SlDML2* and regulate the mRNA stability of *SlDML2* through N^6^-Methyladenosine (m^6^A) demethylation (Zhou et al., 2019). After *SlALKBH2* was knocked out by CRISPR/Cas9 gene editing technology, the fruit ripening process was delayed. These results indicate that there is an intrinsic relationship between RNA methylation and DNA methylation. There is also evidence indicating the participation of DNA methyltransferases in the regulation of tomato fruit ripening. When knocked out *Solanum lycopersicum* methyltransferase1 (*SlMET1*), most ripening-related RIN target genes were up-regulated, indicating that SlMET1 may repress these genes (Yang et al., 2019). These findings suggest that DNA methylation plays an important role, not in isolation, but in synergy with other signals to regulate tomato ripening (**Figure 1**). Currently, our understanding of the various signaling molecules involved in DNA methylation is not comprehensive enough. Future studies are needed to further enrich this molecular regulatory network.

### 2.2 Histone modification

Histone modification refers to the methylation, acetylation, phosphorylation, and other modifications of the amino terminus of histones (Patel and Wang, 2013). These modifications can alter the state of chromatin and regulate gene expression. Up to now, 52 histone methyltransferases (HMTs), 26 histone demethylases (HDMs), 32 histone acetyltransferases (HATs), and 15 histone deacetylases (HDACs) have been identified in tomato genome (Aiese Cigliano et al., 2013). Relevant investigations have demonstrated the significant contribution of histone methylation to fruit ripening. Plant development is profoundly shaped by the pivotal functions executed by Polycomb group (PcG) proteins, which epigenetically suppress the transcription of target genes.Overexpression of *SlMSI1* (*Solanum lycopersicum MULTICOPY SUPPRESSOR OF IRA1*), a component of polycomb repressive complex 2 (PRC2), in the histone H3K27 methylase protein complex in tomato inhibited the expression of ripening related genes and hindered the fruit ripening process (Liu et al., 2016). Similar results were found in PRC1 protein SlLHP1b (*Solanum lycopersicum* Like Heterochromatin Protein 1b). SlLHP1b could bind H3K27me mark in regions of ripening-associated chromatin, targeting ripening-related ethylene biosynthesis, carotenoid biosynthesis and *RIN* targeted genes, repressing fruit ripening (Liang et al., 2020). Meanwhile, SlLHP1b interacted with SlMSI1, suggesting a potential collaborative role of these two proteins in regulating tomato fruit ripening (Liang et al., 2020). Enhancer of Zeste (EZ) Polycomb group protein SlEZ2 was a member of PRC2, reduction of this gene expression resulted in alteration in fruit development and ripening (Boureau et al., 2016). Moreover, genome-wide analysis of H3K27e3 revealed that this epigenetic modification was negatively associated with ripening-related genes expression (Lü et al., 2018). *SlJMJ6* (*Solanum lycopersicum* Jumonji C domain-containing protein 6) encodes a histone lysine H3K27 demethylase, and the overexpression of *SlJMJ6* accelerated the process of fruit ripening (Li et al., 2020). Subsequent investigations revealed that SlJMJ6 could directly target 32 genes, including *RIN*, *SlDML2*, thereby modulating the transcription of these genes. Another study showed that H3K4 demethylase SlJMJ7 modulated tomato fruit ripening by regulating ethylene biosynthesis genes, ripening related transcription factor and DNA demethylation genes expression through H3K4me3 demethylation (Ding et al., 2022). This finding revealed a crosstalk between histone methylation and DNA methylation.

Apart from histone methylation modifications, histone acetylation modifications also participate in the regulation of tomato fruit ripening. It has been discovered that distinct histone deacetylases play varying roles in the regulation of fruit ripening. Silencing of *SlHDA1* (*Solanum lycopersicum histone deacetylase1*), *SlHDA3* and *SlHDT1* (*Solanum lycopersicum HD-tuins1*) accelerated fruit ripening, while the silencing of *SlHDT3* slowed fruit ripening (Guo et al., 2017a; Guo et al., 2017b; Guo et al., 2018; Guo, 2022). In addition, gene knockout experiment revealed that the histone variant Sl_H2A.Z regulated the expression of carotenoids biosynthesis related genes, including *SlPSY1* (*Solanum lycopersicum PHYTOENE SYNTHASE 1*), *SlPDS* (*Solanum lycopersicum PHYTOENE DESATURASE 1*), and *SlVDE* (*Solanum lycopersicum VIOLAXANTHIN DE-EPOXIDASE*) in tomato ripening (Yang et al., 2021). These findings indicate that histone modifications may play a significant regulatory role in tomato fruit ripening. Future research will focus on further identifying the histone modification molecules involved in the regulation of ripening.

### 2.3 Non-coding RNA

In addition to DNA/RNA methylation and histone modification, non-coding RNA also participates in the regulation of tomato fruit ripening. Non-coding RNAs are a class of RNAs that do not code for proteins (Ma et al., 2020). In studies on regulation of fruit ripening, researchers mainly focus on microRNA (miRNA), long non-coding RNA (lncRNA) and circular RNA (circRNA). Researchers performed high-throughput sequencing on tomato fruits and identified a collection of non-coding RNAs that could potentially participate in fruit ripening (Gao et al., 2015; Zhu et al., 2015; Tan et al., 2017). It was found that the expression of 19 conserved microRNAs in fruits of *rin* mutants was significantly different from that of wild types (Gao et al., 2015). Another study analyzed lncRNAs in *rin* mutants and wild-type fruits, and identified 677 differentially expressed lncRNAs (Zhu et al., 2015). Some identified lncRNAs were silenced instantaneously using virus-induced gene silencing technology, leading to a delay in the fruit ripening process. A total of 1018 circRNAs were identified in tomato fruits through deep sequencing of RNA samples, with rRNA and RNase R digestion removed. Notably, some of these circRNAs showed a close correlation with pigment synthesis (Tan et al., 2017). Furthermore, a recent review has synthesized the current findings regarding non-coding RNAs associated with fruit ripening. This review delineated that out of these non-coding RNAs, 40 were linked to the ethylene pathway, 8 to color, 14 to flavor, and 32 to texture in tomato fruit ripening (Ma et al., 2020). In terms of the specific roles of individual miRNA genes, it was reported that upon the knockout of *SlMIR164A* using CRISPR/Cas9 gene editing technology, the fruit ripening process accelerated, and there were alterations in sugar and organic acid contents (Lin et al., 2022). It is evident that non-coding RNAs may have crucial functions in the process of tomato fruit ripening. With further functional verification of non-coding RNAs, researchers will gain a deeper understanding and be able to analyze the mechanisms by which these non-coding RNAs regulate fruit ripening.

## 3 Discussion

The functional analysis of the key components of DNA methylation, RNA methylation, histone modification and non-coding RNA in tomato fruit ripening regulation revealed a complex regulation network (**Figure 1**). Previous study showed that the RNA demethylase SlALKBH2 could regulate the mRNA stability of the DNA demethylase SlDML2 through m^6^A demethylation (Zhou et al., 2019), elucidating the interaction between RNA methylation and DNA methylation in tomato fruit ripening regulation. Meanwhile, the H3K4 demethylase SlJMJ7 could repress the DNA demethylation gene *SlDML2* expression by H3K4me3 demethylation, thus establishing a crosstalk between histone and DNA demethylation in tomato fruit ripening regulation (Ding et al., 2022). An integrated assessment of alterations in genome methylation, long non-coding RNAs, circular RNAs, micro RNAs, and fruit metabolites unveiled numerous differentially expressed genes (DEGs). These DEGs encompass differentially methylated regions that encode transcription factors and pivotal enzymes linked to ethylene or carotenoid pathways, which could potentially be targeted by differentially expressed non-coding RNAs (Zuo et al., 2020). Existing research indicates that tomato fruit ripening regulatory factors do not act in isolation but rather function in coordination. Currently, our understanding of these cooperative mechanisms is not sufficiently deep. Future research can focus on exploring the interactions among DNA or RNA methylation modifications, histone modification, and non-coding RNA, which will give a more comprehensive and intriguing regulatory network for tomato fruit ripening. At present, research efforts are predominantly focused on DNA demethylases, RNA demethylases, histone demethylases, and histone deacetylases. There is relatively less research conducted on DNA methyltransferases, RNA methyltransferases, histone methyltransferases, and histone acetyltransferases. In the future, there should be a strengthening of research in these areas to gain a more comprehensive understanding.

Currently, the online Tomato Epigenome Database (http://ted.bti.cornell.edu/epigenome/) offers a valuable resource for searching DNA methylation patterns and accessing information on cytosine methylation, gene expression, small RNA, and RIN-binding profiles through the Genome Browser. Further development of similar online tools and standalone software is essential to facilitate rapid and convenient investigations into the role of epigenetics in tomato fruit ripening. Additionally, advancements in sequencing and experimental technologies, such as 3D genome analysis, single-cell sequencing, and spatial-temporal omics, will enable a more extensive and in-depth exploration of the epigenetic regulation within the tomato fruit ripening regulatory network in the future.

## Author contributions

YM: Writing – original draft, Writing – review & editing. LJ: Writing – original draft, Writing – review & editing. DJ: Conceptualization, Investigation, Project administration, Visualization, Writing – original draft, Writing – review & editing.

## References

[B1] Aiese Cigliano R. Sanseverino W. Cremona G. Ercolano M. R. Conicella C. Consiglio F. M. (2013). Genome-wide analysis of histone modifiers in tomato: gaining an insight into their developmental roles. BMC Genomics 14, 57. doi: 10.1186/1471-2164-14-57 23356725 PMC3567966

[B2] Bemer M. Karlova R. Ballester A. R. Tikunov Y. M. Bovy A. G. Wolters-Arts M.. (2012). The tomato fruitfull homologs TDR4/FUL1 and MBP7/FUL2 regulate ethylene-independent aspects of fruit ripening. Plant Cell 24, 4437–4451. doi: 10.1105/tpc.112.103283 23136376 PMC3531844

[B3] Boureau L. How-Kit A. Teyssier E. Drevensek S. Rainieri M. Joubès J.. (2016). A CURLY LEAF homologue controls both vegetative and reproductive development of tomato plants. Plant Mol. Biol. 90, 485–501. doi: 10.1007/s11103-016-0436-0 26846417

[B4] Chen T. Qin G. Tian S. (2020). Regulatory network of fruit ripening: current understanding and future challenges. New Phytol. 228, 1219–1226. doi: 10.1111/nph.16822 32729147

[B5] Ding X. Liu X. Jiang G. Li Z. Song Y. Zhang D.. (2022). SlJMJ7 orchestrates tomato fruit ripening via crosstalk between H3K4me3 and DML2-mediated DNA demethylation. New Phytol. 233, 1202–1219. doi: 10.1111/nph.17838 34729792

[B6] Eichten S. R. Schmitz R. J. Springer N. M. (2014). Epigenetics: beyond chromatin modifications and complex genetic regulation. Plant Physiol. 165, 933–947. doi: 10.1104/pp.113.234211 24872382 PMC4081347

[B7] Eriksson E. M. Bovy A. Manning K. Harrison L. Andrews J. De Silva J.. (2004). Effect of the *Colorless non-ripening* mutation on cell wall biochemistry and gene expression during tomato fruit development and ripening. Plant Physiol. 136, 4184–4197. doi: 10.1104/pp.104.045765 15563627 PMC535848

[B8] Fujisawa M. Nakano T. Ito Y. (2011). Identification of potential target genes for the tomato fruit-ripening regulator RIN by chromatin immunoprecipitation. BMC Plant Biol. 11, 26. doi: 10.1186/1471-2229-11-26 21276270 PMC3039564

[B9] Fujisawa M. Shima Y. Nakagawa H. Kitagawa M. Kimbara J. Nakano T.. (2014). Transcriptional regulation of fruit ripening by tomato FRUITFULL homologs and associated MADS box proteins. Plant Cell 26, 89–101. doi: 10.1105/tpc.113.119453 24415769 PMC3963596

[B10] Gao C. Ju Z. Cao D. Zhai B. Qin G. Zhu H.. (2015). MicroRNA profiling analysis throughout tomato fruit development and ripening reveals potential regulatory role of RIN on microRNAs accumulation. Plant Biotechnol. J. 13, 370–382. doi: 10.1111/pbi.12297 25516062

[B11] Gao Y. Zhu N. Zhu X. Wu M. Jiang C.-Z. Grierson D.. (2019). Diversity and redundancy of the ripening regulatory networks revealed by the fruitENCODE and the new CRISPR/Cas9 *CNR* and *NOR* mutants. Hortic. Res. 6, 39. doi: 10.1038/s41438-019-0122-x 30774962 PMC6370854

[B12] Gapper N. E. McQuinn R. P. Giovannoni J. J. (2013). Molecular and genetic regulation of fruit ripening. Plant Mol. Biol. 82, 575–591. doi: 10.1007/s11103-013-0050-3 23585213

[B13] Giovannoni J. (2001). Molecular regulation of fruit ripening. Annu. Rev. Plant Physiol. Plant Mol. Biol. 52, 725–749. doi: 10.1146/annurev.arplant.52.1.725 11337414

[B14] Giovannoni J. J. (2004). Genetic regulation of fruit development and ripening. Plant Cell 16, 170–181. doi: 10.1105/tpc.019158 PMC2643394 15010516

[B15] Giovannoni J. Nguyen C. Ampofo B. Zhong S. Fei Z. (2017). The epigenome and transcriptional dynamics of fruit ripening. Annu. Rev. Plant Biol. 68, 61–84. doi: 10.1146/annurev-arplant-042916-040906 28226232

[B16] Guo J.-E. (2022). Histone deacetylase gene *SlHDT1* regulates tomato fruit ripening by affecting carotenoid accumulation and ethylene biosynthesis. Plant Sci. 318, 111235. doi: 10.1016/j.plantsci.2022.111235 35351307

[B17] Guo J.-E. Hu Z. Li F. Zhang L. Yu X. Tang B.. (2017a). Silencing of histone deacetylase *SlHDT3* delays fruit ripening and suppresses carotenoid accumulation in tomato. Plant Sci. 265, 29–38. doi: 10.1016/j.plantsci.2017.09.013 29223340

[B18] Guo J.-E. Hu Z. Yu X. Li A. Li F. Wang Y.. (2018). A histone deacetylase gene, *SlHDA3*, acts as a negative regulator of fruit ripening and carotenoid accumulation. Plant Cell Rep. 37, 125–135. doi: 10.1007/s00299-017-2211-3 28932910

[B19] Guo J.-E. Hu Z. Zhu M. Li F. Zhu Z. Lu Y.. (2017b). The tomato histone deacetylase SlHDA1 contributes to the repression of fruit ripening and carotenoid accumulation. Sci. Rep. 7, 7930. doi: 10.1038/s41598-017-08512-x 28801625 PMC5554242

[B20] He L. Huang H. Bradai M. Zhao C. You Y. Ma J.. (2022). DNA methylation-free *Arabidopsis* reveals crucial roles of DNA methylation in regulating gene expression and development. Nat. Commun. 13, 1335. doi: 10.1038/s41467-022-28940-2 35288562 PMC8921224

[B21] Itkin M. Seybold H. Breitel D. Rogachev I. Meir S. Aharoni A. (2009). TOMATO AGAMOUS-LIKE1 is a component of the fruit ripening regulatory network. Plant J. 60, 1081–1095. doi: 10.1111/j.1365-313X.2009.04064.x 19891701

[B22] Ito Y. Kitagawa M. Ihashi N. Yabe K. Kimbara J. Yasuda J.. (2008). DNA-binding specificity, transcriptional activation potential, and the *rin* mutation effect for the tomato fruit-ripening regulator RIN. Plant J. 55, 212–223. doi: 10.1111/j.1365-313X.2008.03491.x 18363783

[B23] Ji D. Cui X. Qin G. Chen T. Tian S. (2020). SlFERL interacts with S-adenosylmethionine synthetase to regulate fruit ripening. Plant Physiol. 184, 2168–2181. doi: 10.1104/pp.20.01203 32999005 PMC7723100

[B24] Ji Y. Wang A. (2023). Recent advances in epigenetic triggering of climacteric fruit ripening. Plant Physiol. 192, 1711–1717. doi: 10.1093/plphys/kiad206 37002826 PMC10315304

[B25] Lang Z. Wang Y. Tang K. Tang D. Datsenka T. Cheng J.. (2017). Critical roles of DNA demethylation in the activation of ripening-induced genes and inhibition of ripening-repressed genes in tomato fruit. Proc. Natl. Acad. Sci. U.S.A. 114, E4511–E4519. doi: 10.1073/pnas.1705233114 28507144 PMC5465898

[B26] Li S. Chen K. Grierson D. (2019). A critical evaluation of the role of ethylene and MADS transcription factors in the network controlling fleshy fruit ripening. New Phytol. 221, 1724–1741. doi: 10.1111/nph.15545 30328615

[B27] Li Z. Jiang G. Liu X. Ding X. Zhang D. Wang X.. (2020). Histone demethylase SlJMJ6 promotes fruit ripening by removing H3K27 methylation of ripening-related genes in tomato. New Phytol. 227, 1138–1156. doi: 10.1111/nph.16590 32255501

[B28] Li X. Wang X. Zhang Y. Zhang A. You C.-X. (2022). Regulation of fleshy fruit ripening: from transcription factors to epigenetic modifications. Hortic. Res. 9, uhac013. doi: 10.1093/hr/uhac013 35147185 PMC9035223

[B29] Li L. Zhu B. Yang P. Fu D. Zhu Y. Luo Y. (2011). The regulation mode of RIN transcription factor involved in ethylene biosynthesis in tomato fruit. J. Sci. Food Agric. 91, 1822–1828. doi: 10.1002/jsfa.4390 21520447

[B30] Liang Q. Deng H. Li Y. Liu Z. Shu P. Fu R.. (2020). Like Heterochromatin Protein 1b represses fruit ripening via regulating the H3K27me3 levels in ripening-related genes in tomato. New Phytol. 227, 485–497. doi: 10.1111/nph.16550 32181875

[B31] Lin D. Zhu X. Qi B. Gao Z. Tian P. Li Z.. (2022). *SlMIR164A* regulates fruit ripening and quality by controlling *SlNAM2* and *SlNAM3* in tomato. Plant Biotechnol. J. 20, 1456–1469. doi: 10.1111/pbi.13824 35403821 PMC9342619

[B32] Liu R. How-Kit A. Stammitti L. Teyssier E. Rolin D. Mortain-Bertrand A.. (2015). A DEMETER-like DNA demethylase governs tomato fruit ripening. Proc. Natl. Acad. Sci. U.S.A. 112, 10804–10809. doi: 10.1073/pnas.1503362112 26261318 PMC4553810

[B33] Liu M. Pirrello J. Chervin C. Roustan J. P. Bouzayen M. (2015). Ethylene control of fruit ripening: revisiting the complex network of transcriptional regulation. Plant Physiol. 169, 2380–2390. doi: 10.1104/pp.15.01361 26511917 PMC4677914

[B34] Liu Z. Wu X. Liu H. Zhang M. Liao W. (2022). DNA methylation in tomato fruit ripening. Physiol. Plantarum 174, e13627. doi: 10.1111/ppl.13627 35040145

[B35] Liu D.-D. Zhou L.-J. Fang M.-J. Dong Q.-L. An X.-H. You C.-X.. (2016). Polycomb-group protein SlMSI1 represses the expression of fruit-ripening genes to prolong shelf life in tomato. Sci. Rep. 6, 31806. doi: 10.1038/srep31806 27558543 PMC4997261

[B36] Lü P. Yu S. Zhu N. Chen Y. R. Zhou B. Pan Y.. (2018). Genome encode analyses reveal the basis of convergent evolution of fleshy fruit ripening. Nat. Plants 4, 784–791. doi: 10.1038/s41477-018-0249-z 30250279

[B37] Ma L. Mu J. Grierson D. Wang Y. Gao L. Zhao X.. (2020). Noncoding RNAs: functional regulatory factors in tomato fruit ripening. Theor. Appl. Genet. 133, 1753–1762. doi: 10.1007/s00122-020-03582-4 32211918

[B38] Manning K. Tör M. Poole M. Hong Y. Thompson A. J. King G. J.. (2006). A naturally occurring epigenetic mutation in a gene encoding an SBP-box transcription factor inhibits tomato fruit ripening. Nat. Genet. 38, 948–952. doi: 10.1038/ng1841 16832354

[B39] Martel C. Vrebalov J. Tafelmeyer P. Giovannoni J. J. (2011). The tomato MADS-box transcription factor RIPENING INHIBITOR interacts with promoters involved in numerous ripening processes in a COLORLESS NONRIPENING-dependent manner. Plant Physiol. 157, 1568–1579. doi: 10.1104/pp.111.181107 21941001 PMC3252172

[B40] Mcmurchie E. J. Mcglasson W. B. Eaks I. L. (1972). Treatment of fruit with propylene gives information about the biogenesis of ethylene. Nature 237, 235–236. doi: 10.1038/237235a0 4557321

[B41] Niu Q. Xu Y. Huang H. Li L. Tang D. Wu S.. (2022) Two MADS-box transcription factors mediate epigenetic control of tomato fruit ripening (Research Square). Available at: https://www.researchsquare.com/article/rs-2003863/v1 (Accessed 22 June, 2023).

[B42] Patel D. J. Wang Z. (2013). Readout of epigenetic modifications. Annu. Rev. Biochem. 82, 81–118. doi: 10.1146/annurev-biochem-072711-165700 23642229 PMC4696766

[B43] Qin G. Wang Y. Cao B. Wang W. Tian S. (2012). Unraveling the regulatory network of the MADS box transcription factor RIN in fruit ripening. Plant J. 70, 243–255. doi: 10.1111/j.1365-313X.2011.04861.x 22098335

[B44] Qin G. Zhu Z. Wang W. Cai J. Chen Y. Li L.. (2016). A tomato vacuolar invertase inhibitor mediates sucrose metabolism and influences fruit ripening. Plant Physiol. 172, 1596–1611. doi: 10.1104/pp.16.01269 27694342 PMC5100769

[B45] Shinozaki Y. Nicolas P. Fernandez-Pozo N. Ma Q. Evanich D. J. Shi Y.. (2018). High-resolution spatiotemporal transcriptome mapping of tomato fruit development and ripening. Nat. Commun. 9, 364. doi: 10.1038/s41467-017-02782-9 29371663 PMC5785480

[B46] Smaczniak C. Immink R. G. H. Angenent G. C. Kaufmann K. (2012). Developmental and evolutionary diversity of plant MADS-domain factors: insights from recent studies. Development 139, 3081–3098. doi: 10.1242/dev.074674 22872082

[B47] Tan J. Zhou Z. Niu Y. Sun X. Deng Z. (2017). Identification and functional characterization of tomato circRNAs derived from genes involved in fruit pigment accumulation. Sci. Rep. 7, 8594. doi: 10.1038/s41598-017-08806-0 28819222 PMC5561264

[B48] Vrebalov J. Pan I. L. Arroyo A. J. M. McQuinn R. Chung M. Poole M.. (2009). Fleshy fruit expansion and ripening are regulated by the tomato SHATTERPROOF gene *TAGL1* . Plant Cell 21, 3041–3062. doi: 10.1105/tpc.109.066936 19880793 PMC2782289

[B49] Vrebalov J. Ruezinsky D. Padmanabhan V. White R. Medrano D. Drake R.. (2002). A MADS-box gene necessary for fruit ripening at the tomato ripening-inhibitor (*rin*) locus. Science 296, 343–346. doi: 10.1126/science.1068181 11951045

[B50] Wang Y. Wang W. Cai J. Zhang Y. Qin G. Tian S. (2014). Tomato nuclear proteome reveals the involvement of specific E2 ubiquitin-conjugating enzymes in fruit ripening. Genome Biol. 15, 548. doi: 10.1186/s13059-014-0548-2 25464976 PMC4269173

[B51] Yang Y. Tang K. Datsenka T. U. Liu W. Lv S. Lang Z.. (2019). Critical function of DNA methyltransferase 1 in tomato development and regulation of the DNA methylome and transcriptome. J. Integr. Plant Biol. 61, 1224–1242. doi: 10.1111/jipb.12778 30652405

[B52] Yang X. Zhang X. Yang Y. Zhang H. Zhu W. Nie W.-F. (2021). The histone variant Sl_H2A.Z regulates carotenoid biosynthesis and gene expression during tomato fruit ripening. Hortic. Res. 8, 85. doi: 10.1038/s41438-021-00520-3 33790255 PMC8012623

[B53] Zhang H. Lang Z. Zhu J.-K. (2018). Dynamics and function of DNA methylation in plants. Nat. Rev. Mol. Cell Biol. 19, 489–506. doi: 10.1038/s41580-018-0016-z 29784956

[B54] Zhong S. Fei Z. Chen Y. R. Zheng Y. Huang M. Vrebalov J.. (2013). Single-base resolution methylomes of tomato fruit development reveal epigenome modifications associated with ripening. Nat. Biotechnol. 31, 154–159. doi: 10.1038/nbt.2462 23354102

[B55] Zhou L. Tian S. Qin G. (2019). RNA methylomes reveal the m^6^A-mediated regulation of DNA demethylase gene *SlDML2* in tomato fruit ripening. Genome Biol. 20, 156. doi: 10.1186/s13059-019-1771-7 31387610 PMC6683476

[B56] Zhu B. Yang Y. Li R. Fu D. Wen L. Luo Y.. (2015). RNA sequencing and functional analysis implicate the regulatory role of long non-coding RNAs in tomato fruit ripening. EXBOTJ 66, 4483–4495. doi: 10.1093/jxb/erv203 PMC4507755 25948705

[B57] Zuo J. Grierson D. Courtney L. T. Wang Y. Gao L. Zhao X.. (2020). Relationships between genome methylation, levels of non-coding RNAs, mRNAs and metabolites in ripening tomato fruit. Plant J. 103, 980–994. doi: 10.1111/tpj.14778 32314448

